# Evaluation of Tomato Germplasm against Tomato Brown Rugose Fruit Virus and Identification of Resistance in *Solanum pimpinellifolium*

**DOI:** 10.3390/plants13050581

**Published:** 2024-02-21

**Authors:** Namrata Jaiswal, Bidisha Chanda, Andrea Gilliard, Ainong Shi, Kai-Shu Ling

**Affiliations:** 1 United States Department of Agriculture, Agricultural Research Service, U.S. Vegetable Laboratory, Charleston, SC 29414, USA; namrata.jaiswal@usda.gov (N.J.); bidisha.chanda@gmail.com (B.C.); andrea.gilliard@usda.gov (A.G.); 2 Department of Horticulture, University of Arkansas, Fayetteville, AR 72701, USA; ashi@uark.edu

**Keywords:** tobamoviruses, tomato brown rugose fruit virus (ToBRFV), genetic resistance, tomato, *Solanum lycopersicum*

## Abstract

The tomato is one of the most important vegetable crops grown worldwide. Tomato brown rugose fruit virus (ToBRFV), a seed-borne tobamovirus, poses a serious threat to tomato production due to its ability to break the resistant genes (*Tm-1*, *Tm-2*, *Tm-2^2^*) in tomatoes. The objective of this work was to identify new resistant source(s) of tomato germplasm against ToBRFV. To achieve this aim, a total of 476 accessions from 12 *Solanum* species were tested with the ToBRFV US isolate for their resistance and susceptibility. As a result, a total of 44 asymptomatic accessions were identified as resistant/tolerant, including thirty-one accessions of *S. pimpinellifolium*, one accession of *S. corneliomulleri*, four accessions of *S. habrochaites*, three accessions of *S. peruvianum,* and five accessions of *S.* subsection *lycopersicon* hybrid. Further analyses using serological tests identified four highly resistant *S. pimpinellifolium* lines, PI 390713, PI 390714, PI 390716, and PI 390717. The inheritance of resistance in the selected lines was verified in the next generation and confirmed using RT-qPCR. To our knowledge, this is a first report of high resistance to ToBRFV in *S. pimpinellifolium*. These new genetic resources will expand the genetic pool available for breeders to develop new resistant cultivars of tomato against ToBRFV.

## 1. Introduction

The tomato (*Solanum lycopersicum* L.) is one of the most important vegetable crops worldwide. In the last few years, the tomato industry has faced a serious threat by the emerging tomato brown rugose fruit virus (ToBRFV), a seed-borne tobamovirus causing disease outbreaks to tomato productions in many countries around the world [1,2]. This emerging tobamovirus was first discovered to infect tomatoes in Jordan and Israel in 2014–2015 [3,4]. Since then, disease outbreaks caused by ToBRFV have been reported in at least 25 countries in five continents, including Asia [5,6,7,8,9,10], Africa [11], Europe [12,13,14,15,16,17,18,19,20,21,22], North America [23,24,25,26,27], and South America [28]. A handful of other countries also reported outbreaks through the European and Mediterranean Plant Protection Organization [29]. Thus, ToBRFV has been considered a global pandemic for the tomato and pepper [1,2]. The rapid spread of ToBRFV outbreaks around the globe is likely caused by several factors including seed-borne outbreaks, mechanical transmission, and resistance breaking to the popular *Tm-2^2^* gene in tomatoes, as well as increasing the off-shore commercial seed production and global trade activities of seed and produce. The potential dire consequences of ToBRFV on tomato and pepper has prompted many countries to impose quarantine status to ToBRFV [2]. In the United States, the USDA-APHIS issued a Federal Order in 2019 to inspect imported tomato and pepper seeds and produce from selected countries with ToBRFV.

With few options available for viral disease management, planting a disease-resistant cultivar would be the most economic and eco-friendly measure for ToBRFV disease management. Several resistant genes (*Tm-1*, *Tm-2* and *Tm-2^2^*) have been used for tomato breeding to control tobamoviruses for tomatoes [30,31,32]. The *Tm-1* gene was derived from *S. habrochaites* PI 126445, the *Tm-2* from *S. peruvianum* PI 126926, and the *Tm-2^2^* gene from *S. peruvianum* PI 128650 [1]. Although *Tm-2* and *Tm-2^2^* are allelic, the *Tm-2^2^* gene is the most effective and durable against many strains of tobacco mosaic virus (TMV) and tomato mosaic virus (ToMV) [33,34]. However, the emerging ToBRFV breaks the popular *Tm-2^2^* gene in tomatoes [4,35,36] that has been used in tomato breeding for tobamovirus control over the past 60 years [37]. This resistant breaking ability renders all commercial tomato cultivars vulnerable to ToBRFV infection, necessitating the urgent need to screen tomato germplasm collections for new sources of ToBRFV resistance.

Genetic resistance is one of the most effective approaches to combat the emerging disease caused by ToBRFV. The term ‘tolerance’ to diseases describes a plant’s defense of exerting no direct impact on the pathogen, but having minimal to no apparent effect on the health and fitness of the host upon infection. In the present study, it is defined as a plant showing no symptoms despite being infected with the virus [38]. The term ‘resistance’ is an infected plant showing no symptoms and also having a reduced virus titer from a systemic infection in comparison to a closely related control plant [39,40]. The term ‘immunity’ to diseases has a broad definition for a host plant to prevent the development of a pathogen or counteract the effect of its products. In the present study, it is defined as an inoculated plant showing no symptoms and testing negative for the virus [41]. Several efforts have been made in the search for new sources of genetic resistance to ToBRFV [42,43,44,45]. There is certainly remarkable complexity in the genetic resistance to ToBRFV, with more *Solanum* species in tolerance and few in resistance. These tolerant lines include *S. chilense* [43,45], *S. lycopersicum* [42], *S. lycopersicum* var. cerasiforme [43], *S. ochranthum* [43,44], *S. penellii* [45], *S. peruvianum* [43,44], *S. pimpinellifolium* [42,43,45], and *S. habrochaites* [43,44]. There is little public information available on genetic inheritance, quantitative trait loci (QTLs) analyses and molecular marker development [42].

To characterize the genetic complexity of resistance to ToBRFV in tomato, the objective of the present study was to conduct a large-scale screening of two core collections of tomato germplasm maintained at the Tomato Genetics Resource Center (TGRC) in the University of California Davis and the USDA Plant Genetic Resource Unit in Geneva, NY, for their resistance against the ToBRFV US isolate. The outcomes of the present study would supply novel genetic materials for genetic study and genomic analysis for molecular markers’ development to accelerate tomato breeding for resistance to ToBRFV.

## 2. Results

### 2.1. Primary Screening of USDA and TGRC Tomato Core Germplasm Collections for Resistance to ToBRFV

**Figure 1:**
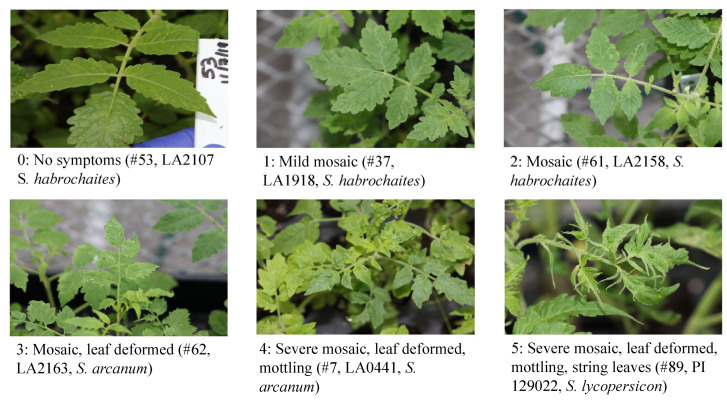
Symptom classes of ToBRFV infection in tomato germplasm used to calculate a disease severity index (DSI). 0: no symptoms; 1: mild mosaic; 2: mosaic; 3: mosaic, leaf deformed; 4: severe mosaic, leaf deformed, mottling; and 5: severe mosaic, leaf deformed, mottling, and string leaves.

The present project was initiated in November 2019 to screen the tomato core collections from USDA and TGRC for their resistance to ToBRFV. A total of 476 tomato accessions were completed, including 86 accessions from TGRC and 390 accessions from USDA. The first experiment was conducted using TGRC materials. Among 86 lines, 3 did not germinate, all others yielded 1–12 seedlings (average 7), which were used for virus inoculation. To minimize any potential escape, all test seedlings were inoculated twice (one week apart). The first symptom reading was conducted at 5 weeks after the first inoculation [36]. The second symptom reading for confirmation was conducted 8 weeks after inoculation. In addition to visual readings of symptom expression on the test plants, leaf tissues from systemic leaves were collected for lab testing using a TMV enzyme-linked immunosorbent assay (ELISA) kit (Agdia, Elkhart, IN, USA) that is known to cross react serologically with other tobamoviruses infecting tomatoes, including ToBRFV [36]. A combination of the symptom expression (0: no symptoms; 1: mild mosaic; 2: mosaic; 3: mosaic, leaf deformed; 4: severe mosaic, leaf deformed, mottling; and 5: severe mosaic, leaf deformed, mottling, and string leaves) and the absorbance readings in ELISA was used to determine resistance (asymptomatic with low to nondetectable ELISA absorbance readings), tolerance (asymptomatic with high ELISA absorbance readings), and susceptibility (mild mosaic to severe shoe-string leaves and higher ELISA readings) (Figure 1; Supplementary Table S1). Based on these criteria, there was no resistant line identified in the TGRC materials, but one accession of *S. habrochaites* (LA2107) was considered tolerance to ToBRFV (Table 1). The remaining 82 accessions were susceptible to ToBRFV (Supplementary Table S1).

The second experiment was conducted in March 2020 using 390 Plant Introductions (PIs) collected from the USDA Plant Genetic Resources Unit in Geneva, NY. Among them, 14 lines did not germinate. For the 376 germinated lines, 1–13 seedlings (average 8) per line were tested for their resistance to ToBRFV. Due to the large number of plants tested in this experiment, we rated disease symptoms carefully from 0 to 5 (Figure 1) to generate a disease severity index (DSI) for each line. A DSI value that was lower than <20% was considered tolerant to ToBRFV (Table 2). Those lines with DSI values that were >20% were considered susceptible to ToBRFV (Supplementary Table S2). Based on these criteria, 43 PIs from five *Solanum* species, including one accession of *S. corneliomulleri* (PI 129144), three accessions of *S. habrochaites* (PI 126445, PI 126445, and PI 247087), three accessions of *S. peruvianum* (PI 306811, PI 390667, PI 390671), and thirty-one accessions of *S. pimpinellifolium* (PI 127805, PI 143524, PI 143527, PI 211838, PI 230327, PI 344102, PI 344103, PI 346340, PI 390692, PI 390693, PI 390694, PI 390695, PI 390698, PI 390699, PI 390700, PI 390702, PI 390710, PI 390712, PI 390713, PI 390714, PI 390716, PI 390717, PI 390720, PI 390722, PI 390723, PI 390724, PI 390725, PI 390726, PI 390727, PI 390750, and PI 432362), and five accessions of *S.* subsection *lycopersicon* hybrid (PI 127799, PI 129143, PI 143522, PI 233930, and PI 237640), were identified as tolerant to ToBRFV (Table 2, Supplementary Table S2). Besides those 43 PIs with tolerance, 333 accessions tested were susceptible to ToBRFV (Supplementary Table S2).

These two preliminary screenings resulted in the identification of 44 accessions with tolerance to ToBRFV, with 43 USDA, and one TGRC tomato accessions (Table 2, Supplementary Tables S1 and S2). From those resistant accessions, one *S. habrochaites*, three *S. lycopersicon* hybrid, one *S. peruvianum*, and eight *S. pimpinellifolium* accessions were still segregating (those lines with 18% > DSI > 3.4% in Table 2).

### 2.2. Rescreening of Selected Lines to Verify Their Resistant Properties to ToBRFV

**Figure 2:**
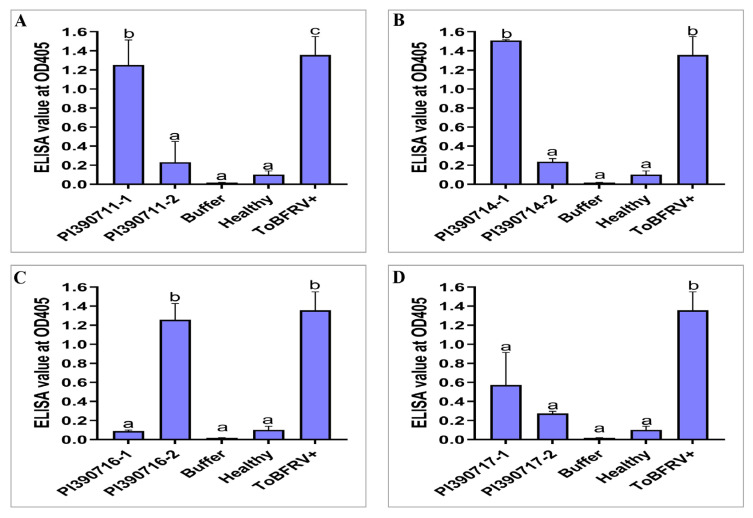
Re-evaluating individual plants from selected *Solanum pimpinellifolium* lines confirmed their resistance to ToBRFV but also revealed a segregating population. Absorbance values at OD_405nm_ for two individual plants derived from seeds generated from self-pollination (S_1_) of four putative ToBRFV-resistant lines, (**A**) PI 390711 (line 326); (**B**) PI 390714 (line 329); (**C**) PI 390716 (line 331); and (**D**) PI 390717 (line 332) along with buffer, healthy tomato, and ToBRFV-infected tomato as controls for comparisons. Data (absorbance readings measured at OD_405nm_) represent mean ± SEM for three experimental replicates. Statistical significance was analyzed using ordinary one-way ANOVA/Dunnette’s multiple comparison followed by Bonferroni post hoc test. Different letters indicate the statistical significance compared with buffer inoculation as mock control. Statistical analysis was performed using GraphPad Prism 9.

**Figure 3:**
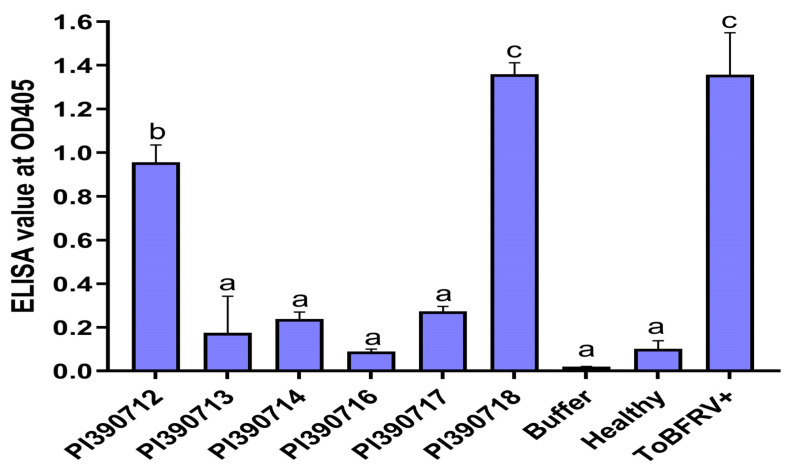
Re-evaluation of selected *Solanum pimpinellifolium* lines for their resistance to ToBRFV using an ELISA test to assess virus titers on leaf tissue samples collected from systemic leaves. Based on statistical analysis, plants from four lines were considered resistant (PI 390713, PI 390714, PI 390716, and PI 390717), whereas plants from two other lines (PI 390712 and PI 390718) were susceptible to ToBRFV. Buffer, healthy tomato, and ToBRFV-infected tomato were included in the same ELISA test as controls for comparison. Data (absorbance readings measured at OD_405 nm_) represent mean ± SEM for three experimental replicates. Statistical significance was analyzed using ordinary one-way ANOVA/Dunnette’s multiple comparison followed by Bonferroni post hoc test. Different letters indicate the statistical significance compared with buffer inoculation as mock control. Statistical analysis was performed using GraphPad Prism 9.

Through self-pollination, single plants from those lines with resistance/tolerance to ToBRFV were advanced to a new generation (S_1_). To assess whether the identified resistance was inheritable to a new generation, we germinated S_1_ seeds from five selected *S. pimpinellifolium* lines with putative resistance/tolerance (line 327: PI 390712; line 328: PI 390713; line 329: PI 390714; line 331: PI 390716; line 332: PI 390717) or one susceptible line (line 333: PI 390718) (Supplementary Table S2) and tested S_1_ seedlings for their responses to ToBRFV infection. Interestingly, our re-test results verified the resistance properties for line 328: PI 390713, with three other lines (line 329: PI 390714, line 331: PI 390716 and line 332: PI 390717) still segregating for their resistance to ToBRFV, as assessed using the ELISA absorbance values with a threshold absorbance level (OD_405nm_ = 0.31) for resistance on individual plants (Figure 2). When ELISA readings from individual plants were combined, the mean absorbance readings for four resistant lines (line 328: PI 390713, line 329: PI 390714, line 331: PI 390716, and line 332: PI 390717) remained below the threshold (0.31), whereas the two susceptible lines had higher absorbance readings, 0.956 for the line 327: PI 390712 and 1.36 for the line 333: PI 390718, relative to 1.239 for the positive control (ToBRFV+) on tomato ‘Moneymaker’ (Figure 3). As expected, there were genetic impurities observed among individual plants in some germplasm materials. For example, the line 326: PI 390711, which showed segregation for resistance to ToBRFV, with a disease severity index at 48% in the preliminary screening (Supplementary Table S2), was still segregating among individual S_1_ plants (Figure 2A). This would need additional self-pollination and further selection of resistant individuals in advanced generations to stabilize the genetic property of resistance.

**Figure 4:**
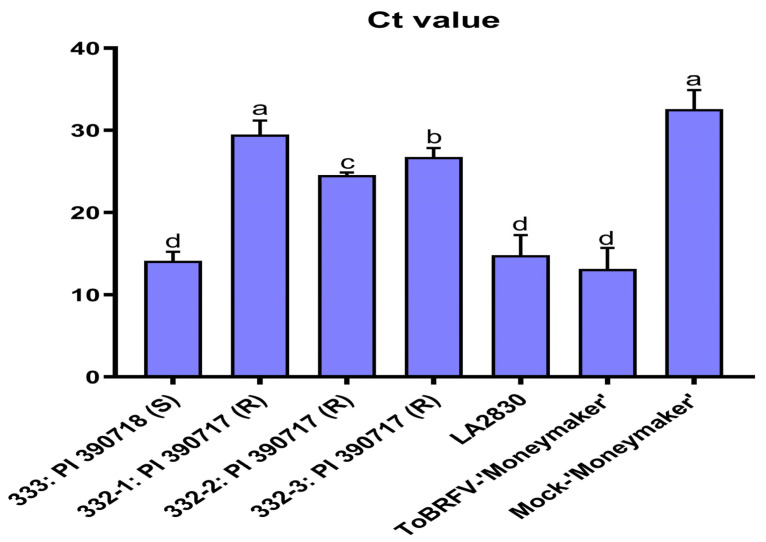
Comparative evaluation on selected tomato lines for their resistance to ToBRFV using RT-qPCR to assess the virus titers on leaf tissue samples collected from systemic leaves. The Ct values from 3 plants (3 biological replicates) of the ToBRFV-resistant line (332-1, 332-2, and 332-3: PI 390717), in comparison to that of the ToBRFV-susceptible line (333: PI 390718), LA2830 (*Tm-1* and *Tm-2^2^*), as well as ToBRFV-infected ‘Moneymaker’, and its mock inoculation control. Data (Ct values) represent mean ± SD for three experimental replicates. Statistical significance was analyzed using ordinary one-way ANOVA/Dunnette’s multiple comparison followed by Bonferroni post hoc test. Different letters indicate the statistical significance compared with buffer inoculation as mock control. Statistical analysis was performed using GraphPad Prism 9.

In addition to using the serological test by ELISA to assess the relative virus titers on tested tomato plants, we also employed a ToBRFV-specific reverse transcription quantitative polymerase chain reaction (RT-qPCR) technology [36] to evaluate the virus titers on ToBRFV-inoculated tomato plants tested in three experimental replicates. While a mean Ct value for the ToBRFV-inoculated tomato ‘Moneymaker’ plant was as low as 13.11 (in high virus titer), a similar low Ct value (14.10) was also observed on a ToBRFV-susceptible *S. pimpinellifolium* Line 333 (PI 390718). As expected, a tomato plant containing *Tm-1* and *Tm-2^2^* (LA2830) showed the same low Ct value (14.79) due to the resistance breaking by ToBRFV. In contrary, three ToBRFV-inoculated plants (biological replicates) from the ToBRFV-resistant *S. pimpinellifolium* Line 332 (PI 390717) had high Ct values (29.50, 24.56 and 26.74). In statistical analysis, the high Ct value in plant-1 (29.50) was shown to have no significant difference to that of the mock control with a mean Ct value of 32.59, whereas the Ct values from two other plants (24.56 and 26.74) were also very high (in low virus titers) (Figure 4, Supplementary Figure S1).

### 2.3. Testing F_1_ Progenies for Their Resistance to ToBRFV

**Figure 5:**
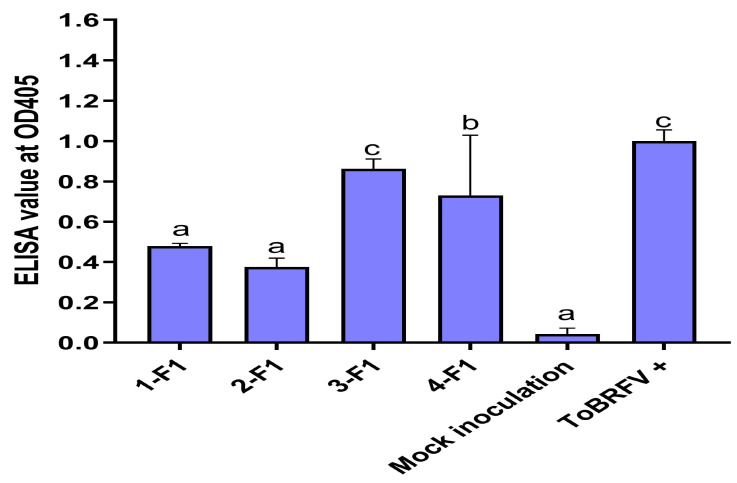
Assessing F_1_ plants derived from selected *Solanum pimpinellifolium* lines for their resistance to ToBRFV as evaluated for their absorbance values (virus titers) using an ELISA test. F_1_ plants from four crosses: 1-F_1_: 327-1 [PI 390712 (S)] × 326 [PI 390711 (R)]; 2-F_1_: 328-1 [PI 390713 (R)] × 326-1 [PI390711 (R)]; 3-F_1_: 333-1 [PI 390718 (S)] × 329-1 [PI 390714 (R)]; and 4-F_1_: 333-1 [PI 390718 (S)] × 332-1 [PI 390717 (R)] were evaluated for their resistance to ToBRFV, in comparison to the mock inoculation control and a positive control (ToBRFV+). Data (absorbance readings measured at OD_405 nm_) represent mean ± SEM for three experimental replicates. Statistical significance was analyzed using ordinary one-way ANOVA/Dunnette’s multiple comparison followed by Bonferroni post hoc test. Different letters indicate the statistical significance compared with buffer inoculation as mock control. Statistical analysis was performed using GraphPad Prism 9.

In addition to evaluating of the self-pollinated seedlings in the S_1_ generation for their resistance to ToBRFV, we also evaluated four F_1_ hybrids generated from crosses between 327-1: PI 390712 (S) × 326: PI 390711 (R), 328-1: PI 390713 (R) × 326-1: PI 390711 (R), 333-1: PI 390718 (S) × 329-1: PI 390714 (R), and 333-1: PI 390718 (S) × 332-1: PI 390717 (R). F_1_ plants derived from all these four crosses were susceptible to ToBRFV, suggesting that the resistance to ToBRFV in *S. pimpinellifolium* is recessive. Although all four F_1_ plants had lower absorbance values (Figure 5) than that of the positive control (1.00), the ELISA readings were still higher than those readings obtained from the S_1_ lines (Figure 3). Since these F_1_ plants were tested in the same ELISA plate as those from the S_1_ plants, their absorbance values were relatively comparable.

## 3. Discussion

In the present study, by screening a total of 476 tomato core accessions from the USDA and TGRC tomato germplasm collections (Table 1, Supplementary Tables S1 and S2), we identified 44 accessions with tolerance to ToBRFV US isolate (Table 2). A large proportion (31 of 44 or 70%) of these tolerant lines belong to *S. pimpinellifolium*. In addition, a number of tolerant lines were also identified from four other species, including *S. corneliomulleri* (1), *S. habrochaites* (4), *S. peruvianum* (3), and *S.* subsection *lycopersicon* hybrid (5) (Table 2). The high genetic diversity of being tolerant/resistant to ToBRFV is in general agreement with the results obtained from earlier studies by other groups using different isolates of ToBRFV [42,43,44,45]. Due to various sources of tomato germplasm collections used for evaluation in the present study, these 44 accessions of tomato germplasm with resistant/tolerant properties to ToBRFV do not overlap with previous studies (Table 2 and Table 3). The reason for these new additions is likely that we focused our efforts mainly on the USDA tomato germplasm collections not previously extensively examined. The smaller number of 86 tomato germplasm accessions from TGRC also had little overlap to those used in previous studies [42,43,44,45].

There is a diversity of genetic sources of tolerance to ToBRFV in tomato germplasm. Several tobamoviruses are known as harmful pathogens to tomato crops. Among them, the emerging resistant breaking ToBRFV has posed a serious threat to profitable tomato productions around the world [1]. However, ToBRFV, a recently emerged plant virus [3], has been shown to infect all known genotypes of the tomato, including those carrying *Tm-1*, *Tm-2*, and *Tm-2^2^* resistance genes [4,36,46]. With no available commercial tomato cultivars with ToBRFV resistance at the moment, growers adopt preventative measures to protect their tomato crops from virus spread in the production greenhouse facilities. Several effective disinfectants have been selected and recommended to growers for virus control [47,48,49,50,51,52,53,54]. However, breeding for disease resistance is still the most powerful and economic way to control viral diseases [55]. Thus, genetic resistance would be the most effective strategy to combat the emerging ToBRFV. Several authors recently reported tolerance/resistance to ToBRFV in genotypes of *S. lycopersicum*, *S. pimpinellifolium*, *S. habrochaites,* and *S. ochrantum* [42,43,44,45]. Although a high number of genetic resources identified with being resistant/tolerant to ToBRFV, most of them are considered as tolerant (asymptomatic) with some levels of virus infection (Table 3).

Because we directly used the seeds that were provided by the germplasm repository for our primary screening, individual plants in certain accessions from the germplasm materials might develop various levels of symptom expression. We considered plants with a disease severity class of less than 1 (or a disease severity index < 20%) as tolerant. In this case, at least one of their plants in an accession should be asymptomatic. For those accessions to be considered as resistant to ToBRFV, in addition to their low disease severity index, some of their plants should also contain a reduced level of virus titer as assessed with ELISA absorbance values, lower than 0.31 (a threshold for resistance) or using RT-qPCR. Through single-plant selection in advanced generations, it is very possible to generate a resistant plant with stable inheritance in genetic resistance to ToBRFV. For the S_1_ generation, two plants per line were tested individually with an ELISA test using leaf tissue samples collected from upper and lower portions of the plant. As shown in Figure 2, the two S_1_ plants tested demonstrated some levels of genetic segregation for resistance in several resistance lines. Therefore, advanced generation through single-plant self-pollination is necessary to obtain lines with stable inheritance of resistance to ToBRFV.

Based on disease severity class, if every plant (average 7–8 plants per accession) developed a mild mosaic symptom in class 1 (DSI 20%), they are considered as susceptible to ToBRFV. If one or more plants in an accession was rated as asymptomatic in class 0, then the DSI will be less than 20%, such as those accessions in DSI numbers (3.4% and 18.0%, Supplementary Table S2). Those accessions would be considered as tolerant to ToBRFV because at least one plant was asymptomatic which could be advanced with single-plant selection through self-pollination. For those accessions to be considered as resistant, some or all of the test plants in an accession would need to produce a significant low level of the virus titer as defined, with a threshold at 0.31, in the absorbance value using an ELISA test. Through advanced generation, those resistant plants with a low absorbance value will be selected for developing a resistant line through self-pollination. Although the line 327 (PI 390712) was rated as asymptomatic in the primary screening (Table 2, Supplementary Table S2), in the S_1_ generation, two plants had higher ELISA readings, which can only be considered as tolerant, but not resistant to ToBRFV (Figure 3). On the other hand, the line 326 (PI 390711) which was rated as susceptible in the preliminary screening (Supplementary Table S2) was segregating for resistance to ToBRFV in S_1_ plants (Table 2) where some resistant individuals could be identified through single-plant selection in advance generations. *S. pimpinellifolium* is a self-compatible species, which can be hand-pollinated using pollen from a flower on the same plant for pollination to generate fruits.

ELISA has been extensively used for virus detection and its OD_405nm_ absorbance values are used for relative quantification of virus titers in infected plant tissue samples when evaluated along with the proper controls (i.e., blank, health tissue, and positive virus-infected controls). In fact, the same ELISA method is used by Zinger et al. 2021 [42] for their evaluation of tomato germplasm materials with resistance to ToBRFV. In the present study, we conducted an extensive screening with a large number of tomato germplasm (476 accessions), each with an average of 7–8 plants, phenotyping with careful symptom observation, and disease severity classes were used to make the initial assessment to identify resistant/tolerant plants. An ELISA test with absorbance values was used only as a secondary to assess relative virus titers. We did not claim immunity for our selected materials, only disease resistance with reduced virus titers in comparison to those readings from the susceptible controls. The low virus titers observed from those resistant lines were confirmed through the use of RT-qPCR on Line 332: PI 390717 (Figure 4).

Nevertheless, several lines of *Solanum* species have been considered as resistant (with no detectable level of the virus based on appropriate laboratory tests, by either ELISA [42] or PCR [43,44]. To decide true resistance, it is necessary to conduct lab tests, using either serological tests [42]) or molecular tests/bioassay [43,44] to determine the presence and concentration of the virus in the systemic tissues. These rigorous tests identified only one source of *S. lycopersicum* [42] and several accessions of *S. habrochaites* and *S. peruvianum* as ToBRFV resistance [44]. The sexual incompatibility between *S. ochranthum* and *S. lycopersicum* limits its utility for tomato breeding [43]. Therefore, the resistant *S. pimpinellifolium* lines identified in this study would offer additional choices of genetic resources likely to be useful for tomato breeding against ToBRFV. Even for an experienced breeder, it is still a challenge to use *S. habrochaites or S. peruvianum* to cross with the tomato (*S. lycopersicum*). *S. pimpinellifolium* is a close relative to *S. lycopersicum*, and the intercross between them is readily compatible in tomato breeding. Therefore, those *S. pimpinellifolium* lines with ToBRFV resistance identified in the present study would offer better genetic materials for breeders to choose in making crosses with their elite tomato lines. Although resistance to ToBRFV in selected *S. pimpinellifolium* is not an immunity, to our knowledge, this is the first report finding true resistance with significant lower virus titers in several *S. pimpinelllifolium* accessions (PI 390713, PI 390714, PI 390716, and PI 390717). This resistance is verified in a separate study (Ling’s lab, unpublished data), where we had used one of the identified *S. pimpinellifolium* lines (PI 390717) to generate F_2_ populations and applied genome re-sequencing technology and quantitative trait locus (QTL) analyses to identify single nucleotide polymorphisms (SNPs) that are associated with the ToBRFV resistance in *S. pimpinellifolium*. Molecular marker technology (i.e., Kompetitive Amplified Specific PCR) will be developed to make the tomato-breeding process using marker-assisted selection for ToBRFV resistance easier.

## 4. Materials and Methods

### 4.1. Plant Germplasm Materials

A total of 476 plant germplasm accessions representing the core collections of *Solanum* species, including 390 Plant Introductions (PIs) from the United States Department of Agriculture (USDA) National Plant Germplasm System (NGPS) and 86 accessions from the Tomato Genetic Resources Center (TGRC) at University of California, Davis was evaluated for their resistance/tolerance to ToBRFV through mechanical inoculation and symptom expression on tomato seedlings. The 12 *Solanum* species and number of accessions used in this study were *S. arcanum* (10); *S. chilense* (17); *S. corneliomulleri* (17); *S. habrochaites* (50); *S. huaylasense* (3); *S. lycopersicum* (11); *Solanum lycopersicum* var. cerasiforme (1); *S. neorickii* (1); *S. pennellii* (1); *S. peruvianum* (73); *S. pimpinellifolium* (140); and *S.* subset. *lycopersicon* hybrid (153) (Table 1). Two experiments were conducted, the first was with 86 accessions of tomato materials from TGRC and the second was with 390 PIs supplied by USDA NPGS. For each accession, 12 seeds were planted individually in a 36-seed starter tray that was filled with soilless growth medium, Metro-Mix 360 (Sun Gro Horticulture, Agawam, MA, USA) for germination in a greenhouse. Most seeds from the germplasm collections were able to germinate, with an average of 7–8 seedings per accession germinated and used for resistance screening.

### 4.2. Virus Culture and Mechanical Inoculation

The ToBRFV US isolate CA18-01 (GenBank Accession No. MT002973; [56]) was collected on a tomato plant from a greenhouse in California [25] and isolated through serial passages on a local lesion host of *Nicotiana tabacum* var. Samsun to obtain a pure culture [36], which was used for this evaluation. We maintained the pure virus culture of ToBRFV on ‘Moneymaker’ tomato plant in an insect-proof BugDorm (BioQuip Products, Compton, CA, USA) in a containment greenhouse with the temperature at 25 °C with 12–14 h natural sunlight. The virus inoculum was prepared by grinding the symptomatic leaves (1:5 *w*/*v*) in a plastic tissue extraction bag containing 1× phosphate-buffered saline solution, pH 7.0 (140 mM NaCl, 8 mM Na_2_HPO_4_, 1.5 mM KH_2_PO_4_, 2.7 mM KCl, and 0.8 mM Na_2_SO_3_) using a Homex-6 tissue homogenizer (Bioreba AG, Reinach, Switzerland). The freshly prepared virus inoculum was kept on ice until it was used. Ten-day-old seedlings (in 2–3 leaf stage) were used for mechanical inoculation. Seedlings were lightly dusted with carborundum (320 grit, ThermoFisher Scientific, Waltham, MA, USA) followed by rub-inoculation as determined in our previous study [36]. The inoculated seedlings were placed under shade for several hours to minimize their potential injury from direct sunlight, then moved and maintained in a containment greenhouse for 4–8 weeks. The symptom expression of the inoculated plants was observed weekly. Both positive and negative controls were included in the screening experiments. The buffer-treated healthy plants were used as a negative control (mock inoculation). Tomato ‘Moneymaker’ plants inoculated with the same ToBRFV culture were used as a positive control. Test plants were visually scored for the presence of symptoms, including mosaic, mottling, necrotic spots, leaf deformation, shoestring leaves, and plant stunting (Figure 1). To confirm the presence or absence of ToBRFV on the test plants, after the final reading on symptoms, a young systemic leaf was collected in a plastic bag and processed for serological testing (enzyme-linked immunosorbent assay, ELISA). To confirm virus infection, a bulk sample consisting of one small leaf from each plant in one line was collected and tested to assess the virus titer (Supplementary Table S1). For those PIs from USDA, only those lines with asymptomatic plants were collected in a bulk per accession and used for an ELISA test. Those test plants from other lines that expressed typical disease symptoms were infected with ToBRFV and, therefore, were not tested.

### 4.3. Virus Detection through a Serological Test Using Enzyme-Linked Immunosorbent Assay

Although a number of the tomato germplasms tested were asymptomatic based on visual observation, we were unsure which ones had true resistance to ToBRFV (low or no detectable absorbance readings in ELISA) or just tolerance (high absorbance readings that were similar to those of symptomatic plants). ELISA was conducted to quantify the virus titer on the inoculated plants in each genotype. We used a commercial ELISA kit for the tobacco mosaic virus (TMV, a tobamovirus with serological cross reaction to ToBRFV) [36] to detect ToBRFV following the manufacturer’s instructions (Agdia, Elkhart, IN, USA). Approximately 200 mg leaf tissue from each sample was collected in an individual plastic bag and homogenized with a Homex6 tissue homogenizer (Bioreba AG, Switzerland) in 4.0 mL of 1 × ELISA general extraction buffer (GEB) (Bioreba AG, Switzerland). Absorbance readings at OD_405 nm_ were quantified using a SpectraMax ELISA microplate reader (Molecular Devices, San Jose, CA, USA). An absorbance value that showed at least twice that of the healthy negative control (buffer inoculated) was considered positive for ToBRFV infection. To determine whether a line is resistant or susceptible to ToBRFV, we evaluated a series of readings to identify an absorbance reading as the threshold level for resistance or susceptibility. Although no detectable readings were seen in systemic leaves from several selected lines, most of the test plants had only some low levels of virus infection in selected resistant lines. An absorbance reading at OD_405 nm_ in less than 0.31 was selected as the threshold for resistance.

### 4.4. Virus Detection Using Reverse Transcription Quantitative Polymerase Chain Reaction (RT-qPCR)

In addition to using the ELISA method to assess the relative virus titers, we also conducted a reverse transcription quantitative polymerase chain reaction (RT-qPCR) as described in detail [36]. Using the Ct values, we could achieve a better understanding of the virus titers on each of the test plants to assess their resistance or susceptibility to ToBRFV. Total plant RNA was extracted from a systemic leaf tissue collected from tomato plants at four weeks post ToBRFV inoculation using a TRIzol reagent following the manufacturer’s instructions (Thermo Fisher Scientific, Gaithersburg, MD, USA). With 500 mg of fresh leaf tissue, in a plastic tissue extraction bag, 2.25 mL of TRIzol reagent (Thermo Fisher Scientific, USA) was added and homogenized thoroughly using a Homex-6 homogenizer (BioReba, Switzerland). The resulting suspension was transferred to a new 2 mL tube, vortexed thoroughly, settled for 5 min at room temperature, and centrifuged at 12,000× *g* for 10 min. After transferring 1.0 mL of clean supernatant to a new tube, 0.4 mL chloroform was added and vortexed vigorously for 15 s, then centrifuged at 12,000× *g* for 15 min at 4 °C. After adding 1.0 mL of isopropyl to a new tube with 600 μL of the supernatant, the mixture was incubated for 10 min at room temperature and the RNA was precipitated with centrifugation at 12,000× *g* for 10 min at 4 °C. After washing twice using 70% ethanol on the pellet, RNA was dissolved in 200 μL of RNAse-free water. To obtain a higher quality of RNA, the raw RNA preparation was ethanol-precipitated one more time, by adding 0.1 volume (22 μL) of 3 M Sodium Acetate and 3 volumes (600 μL) of absolute ethanol. After incubation in −20 °C for 1 h or 4 °C overnight, the mixture was centrifuged at 12,000× *g* for 15 min at 4 °C to obtain the RNA pellet. After washing the RNA pellet using 70% ethanol, the air-dried RNA was dissolved in 100 μL of nuclease-free water, which was used for RT-qPCR. The RT-qPCR was conducted as described in detail in our previous study [36] with the following primers and TaqMan probe (ToBRFV-F1, 5′-GCCCATGGAACTATCAGAAGAA-3′; ToBRFV-R1, 5′-TTCCGGTCTTCGAACGAAAT-3′; ToBRFV-P1, FAM-AGTCCCGATGTCTGTAAGGCTTGC-TAMRA) [36] using a One Step PrimerScript RT-PCR kit following the manufacturer’s instructions (Takara Bio USA, Mountain View, CA, USA). RT-qPCR was carried out on a AriaMX real-time PCR system (Agilent, Santa Clara, CA, USA) using the following thermocycling program: reverse transcription at 50 °C for 30 min, followed by 1 cycle of denaturation at 95 °C for 2 min, and 40 cycles of 95 °C for 10 s and 55 °C for 30 s.

### 4.5. Disease Scoring and Data Analysis

To evaluate the tomato plants with resistance/tolerance to ToBRFV, we conducted replicate experiments through visual observation of symptom expression on each test plant weekly post inoculation for 4–8 weeks. Symptom severities were scored on a 0 to 5 scale, where (0) was no visible symptoms; (1) was mild mosaic; (2) was mosaic; (3) was mosaic with leaf deformed; (4) was severe mosaic with leaf deformed and mottling, and (5) was severe mosaic with deformed leaf, mottling, and shoestring-like leaves (Figure 1).

Disease severity index was calculated using the formula:

$$\mathrm{DSI}\;(\%)=\sum_{i=0}^{5}\frac{iYi\times 100}{5N}$$

DSI (%) = [sum (class frequency × score of rating class)]/[(total number of plants) × (maximal disease index)] × 100

Where *i* = class, *Yi* = number of plants in the class. A disease severity index (DSI) less than 20% was considered tolerance and those with higher DSIs in the 20% to 100% range were considered susceptible to ToBRFV. To be considered resistant, test plants in a germplasm would need to be asymptomatic as well as in lower absorbance (OD_405nm_ at 0.31) to undetectable absorbance readings as tested by ELISA.

### 4.6. Advancing Selected Resistant Lines through Self-Pollination or Cross-Pollination to Generate F_1_ Plants for Evaluation of The Inheritability of Resistance to ToBRFV

To verify the ToBRFV resistant or tolerance from those accessions identified in the preliminary screening of the core tomato germplasms, selected lines were self-pollinated to generate seeds (S_1_). The S_1_ plants from six *S. pimpinellifolium* lines were tested to confirm their resistant properties to ToBRFV. These *S. pimpinellifolium* lines included four highly resistant lines, line 328: PI 390713, line 329: PI 390714, line 331: PI 390716 and line 332: PI390717 and one susceptible line, line 333: PI 390718 from *S. pimpinellifolium*. The resistance properties in two other lines, 326 and 327, between the preliminary screening and the retest using S_1_ plants were not consistent, due to genetic segregation in the germplasm materials. In addition, cross-pollination was conducted to generate F_1_ seeds from selected lines. The F_1_ seedlings generated from selected crosses were evaluated for their resistance to ToBRFV through symptom observation followed by an ELISA test. Some of these crosses will be advanced for more detailed genetic study to characterize the inheritance of resistance and molecular marker development.

### 4.7. Statistical Analysis

All statistical parameters are mentioned in the figure legends. Data represent absorbance readings measured at OD_405 nm_ with mean ± SEM in ELISA or Ct values with mean ± SD in RT-qPCR from three experimental replicates. Statistical significance was analyzed using ordinary one-way ANOVA/Dunnette’s multiple comparison followed by Bonferroni post hoc test. The statistical analysis was performed using GraphPad Prism 9.

## 5. Conclusions

In the present study, we evaluated a total of 476 accessions from 12 *Solanum* species and identified 44 accessions with resistance/tolerance to ToBRFV in five species, including *S. corneliomulleri*, *S. habrochaites*, *S. peruvianum*, *S.* subsec. *lycopersicon* hybrid, and *S. pimpinellifolium*. Upon closer examination and comparison with earlier reported studies, the 44 accessions identified in the present study appeared to be new additions, which enriches the genetic pool for selection in tomato breeding. To our knowledge, this is the first report to identify *S. pimpinellifolium* with true resistance to ToBRFV. The resistant property was verified from at least four accessions of *S. pimpinellifolium* that were originally collected from Peru (USDA GRIN, https://www.ars-grin.gov/, accessed on 2 January 2024). Although it is necessary to follow up on genetic characterization on the inheritance of resistance, a preliminary analysis on the resistance in the F_1_ progenies derived from several *S. pimpinellifolium* crosses proved to be controlled by a recessive gene(s), which appeared to be in general agreement with the result from Zinger et al. 2021 [42]. These ToBRFV-resistant *S. pimpinellifolium* could serve as foundation materials for parents in tomato breeding programs to develop cultivars with ToBRFV resistance, to study the genetic inheritance, and for genomic analyses to develop molecular markers that could be useful for marker-assisted selection.

## Figures and Tables

**Table 1:** Summary of tomato core germplasm in USDA and the Tomato Genetic Resource Center screened for resistance to tomato brown rugose fruit virus.

Species	Total Number of Accessions Screened	Number of Accessions in Resistance/Tolerance
*Solanum arcanum*	10	0
*Solanum chilense*	17	0
*Solanum corneliomulleri*	17	1
*Solanum habrochaites*	50	4
*S. huaylasense*	3	0
*Solanum lycopersicum*	10	0
*Solanum lycopersicum* var. cerasiforme	1	0
*Solanum neorickii*	1	0
*Solanum pennellii*	1	0
*Solanum peruvianum*	73	3
*Solanum pimpinellifolium*	140	31
*Solanum* subsect. *lycopersicon* hybrid	153	5
Total	476	44

**Table 2:** Tomato germplasm with resistance/tolerance to tomato brown rugose fruit virus.

Plant ID	Taxonomy	Disease Severity Index (%)
PI 129144	*Solanum corneliomulleri*	0
PI 126445	*Solanum habrochaites*	17.6
PI 209978	*Solanum habrochaites*	0
PI 247087	*Solanum habrochaites*	0
LA 2107	*Solanum habrochaites*	0
PI 306811	*Solanum peruvianum*	16
PI 390667	*Solanum peruvianum*	0
PI 390671	*Solanum peruvianum*	0
PI 127805	*Solanum pimpinellifolium*	14.2
PI 143524	*Solanum pimpinellifolium*	14.2
PI 143527	*Solanum pimpinellifolium*	0
PI 211838	*Solanum pimpinellifolium*	0
PI 230327	*Solanum pimpinellifolium*	0
PI 344102	*Solanum pimpinellifolium*	0
PI 344103	*Solanum pimpinellifolium*	0
PI 346340	*Solanum pimpinellifolium*	0
PI 390692	*Solanum pimpinellifolium*	0
PI 390693	*Solanum pimpinellifolium*	0
PI 390694	*Solanum pimpinellifolium*	0
PI 390695	*Solanum pimpinellifolium*	0
PI 390698	*Solanum pimpinellifolium*	0
PI 390699	*Solanum pimpinellifolium*	18
PI 390700	*Solanum pimpinellifolium*	0
PI 390702	*Solanum pimpinellifolium*	11.4
PI 390710	*Solanum pimpinellifolium*	0
PI 390712	*Solanum pimpinellifolium*	0
PI 390713	*Solanum pimpinellifolium*	0
PI 390714	*Solanum pimpinellifolium*	0
PI 390716	*Solanum pimpinellifolium*	0
PI 390717	*Solanum pimpinellifolium*	0
PI 390720	*Solanum pimpinellifolium*	8.4
PI 390722	*Solanum pimpinellifolium*	0
PI 390723	*Solanum pimpinellifolium*	16
PI 390724	*Solanum pimpinellifolium*	0
PI 390725	*Solanum pimpinellifolium*	3.4
PI 390726	*Solanum pimpinellifolium*	0
PI 390727	*Solanum pimpinellifolium*	0
PI 390750	*Solanum pimpinellifolium*	0
PI 432362	*Solanum pimpinellifolium*	8.8
PI 127799	*Solanum* subsect. *lycopersicon* hybrid	17.8
PI 129143	*Solanum* subsect. *lycopersicon* hybrid	0
PI 143522	*Solanum* subsect. *lycopersicon* hybrid	18
PI 233930	*Solanum* subsect. *lycopersicon* hybrid	0
PI 237640	*Solanum* subsect. *lycopersicon* hybrid	3.4

**Table 3:** Diversity of germplasm resources with resistance/tolerance to ToBRFV.

	Zinger et al., 2021 [42]	Kabas et al., 2022 [45]	Jewehan et al., 2022a [43]	Jewehan et al., 2022b [44]	This Study
Total lines	160	44	636	173	476
Tolerant lines	*S. pimpinellifolium* (9); *S. Lycopersicum* (8)	*S. pimpinnelifolium* (1); *S. penellii* (1); and *S. chilense* (2)	*S. pimpinelifolium* (26); *S. chilense* (1); *S. lycopersicum* var. cerasiforme (4)		*S. corneliomulleri* (1); *S. habrochaites* (4); *S. peruvianum* (3); *S. pimpinellifolium* (27); and *S.* subsect. *lycopersicon* hybrid (5)
Resistant lines	*S. lycopersicum* (1)		*S. ochrantum* (5)	*S. habrochaites* (9); *S. peruvianum* (1)	*S. pimpinellifolium* (4)

## Data Availability

The datasets presented in this study are available in Tables, Figures, Supplementary Tables, and Supplementary Figures.

## Supplementary Materials

The following supporting information can be downloaded at https://www.mdpi.com/article/10.3390/plants13050581/s1, Supplementary Table S1. Screening the tomato core germplasm collections at TGRC for their resistance to ToBRFV. Supplementary Table S2. Screening the USDA tomato core germplasm collections for resistance to ToBRFV. Supplementary Figure S1. RT-qPCR cycle threshold (Ct) values and their respective graphs.
