# Supplementary figures and images for: Evaluation of Tomato Germplasm against Tomato Brown Rugose Fruit Virus and Identification of Resistance in Solanum pimpinellifolium
Source: Plants (Basel). 2024 Feb 21;13(5):581. doi: 10.3390/plants13050581 (PMC10934377; doi:10.3390/plants13050581)

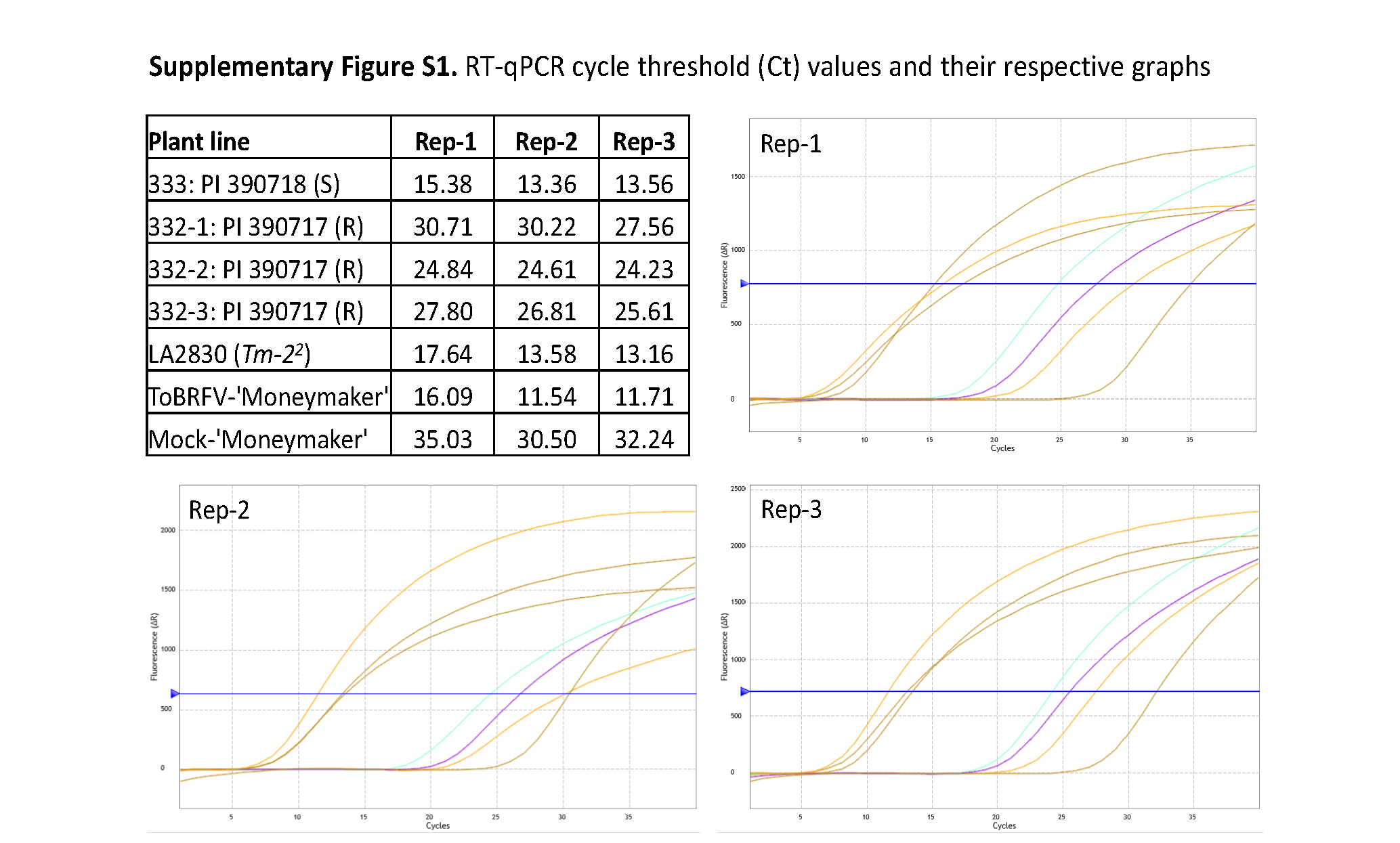

Supplement: Supplementary file 1 [file plants-13-00581-s001.zip › Supplementary Figure S1.tiff]
